# Anderson–Fabry Disease: From Endothelial Dysfunction to Emerging Therapies

**DOI:** 10.1155/2021/5548445

**Published:** 2021-05-13

**Authors:** Cosimo A. Stamerra, Rita Del Pinto, Paolo di Giosia, Claudio Ferri, Amirhossein Sahebkar

**Affiliations:** ^1^University of L'Aquila, Department of Life, Health and Environmental Sciences, Building Delta 6-San Salvatore Hospital, Via Vetoio, Coppito, L'Aquila 67100, Italy; ^2^Biotechnology Research Center, Pharmaceutical Technology Institute, Mashhad University of Medical Sciences, Mashhad, Iran; ^3^Applied Biomedical Research Center, Mashhad University of Medical Sciences, Mashhad, Iran; ^4^School of Pharmacy, Mashhad University of Medical Sciences, Mashhad, Iran

## Abstract

The Anderson–Fabry disease is a rare, X-linked, multisystemic, progressive lysosomal storage disease caused by *α*-galactosidase A total or partial deficiency. The resulting syndrome is mainly characterized by early-onset autonomic neuropathy and life-threatening multiorgan involvement, including renal insufficiency, heart disease, and early stroke. The enzyme deficiency leads to tissue accumulation of the glycosphingolipid globotriaosylceramide and its analogues, but the mechanisms linking such accumulation to organ damage are only partially understood. In contrast, enzyme replacement and chaperone therapies are already fully available to patients and allow substantial amelioration of quality and quantity of life. Substrate reduction, messenger ribonucleic acid (mRNA)-based, and gene therapies are also on the horizon. In this review, the clinical scenario and molecular aspects of Anderson–Fabry disease are described, along with updates on disease mechanisms and emerging therapies.

## 1. Introduction

The Anderson–Fabry disease, or Fabry disease, was described by Johannes Fabry in Germany and William Anderson in England in 1898 [1, 2]. The disease was then attributed to an enzyme defect by Brady in 1967 and Kint in 1970 [3, 4]. It can be defined as an X-linked, multisystemic, progressive lysosomal storage disease, caused by a defect in the GLA gene that encodes for the *α*-galactosidase enzyme (*α*-Gal A) [5]. Over 600 mutations affecting the GLA gene have been described to date [6]. The gene is located on the long arm of the X chromosome (locus Xq22.1), and the disease is transmitted from the mother to the males, hemizygous, and to the females, heterozygous [5]. However, also heterozygous females who inherit the affected gene may manifest the Anderson–Fabry disease, presenting with a rather variable clinical involvement [5]. This phenotypic variability is caused by the phenomenon called “lyonization”: the random inactivation of one of the two X chromosomes of somatic cells. Depending on the number of inactive Xs, the heterozygous woman with Anderson–Fabry disease can present a great variability in the clinical expression of the phenotype, from a condition of total well-being to one characterized by severe symptoms [5]. The partial or complete lack of activity of the enzyme leads to the progressive accumulation of substrate, the glycosphingolipid globotriaosylceramide (GL-3), and its derivative globotriaosylsphingosine (lyso-GL-3), in different cell types. The process preferentially affects the endothelium, myocytes, renal cells, and neurons, increasing the risk of ischemia and tissue infarction [5, 7].

The spectrum of clinical manifestations of Anderson–Fabry disease ranges from the classic to severe phenotype, even in patients with the same genetic mutation [5, 8]. Symptoms of the classic phenotype first occur during childhood or early adolescence with the involvement of the peripheral nervous system. Unlike the classical form, late-onset Fabry disease patients are asymptomatic during childhood or adolescence and become symptomatic between the ages of 30 and 70. Thereafter, disease progression is characterized by renal, cardiac, and neurological involvement, leading to potentially fatal clinical manifestations [8].

Despite a generally more blunted clinical involvement, most of the heterozygous carriers also suffer from significant morbidity and premature mortality [7].

Before the introduction of specific therapy (i.e., enzyme replacement therapy, ERT; oral chaperone therapy), the therapeutic approach to Anderson–Fabry disease essentially consisted of symptomatic treatments, such as the use of analgesics and non-specific measures, including pharmacological prophylaxis of ischemic events, cardiac surgery, dialysis, and renal transplantation.

The purpose of this review is to describe the clinical and molecular aspects of Anderson–Fabry disease, with updates on physiopathological mechanisms and emerging therapies. We performed a review of the available literature in “PubMed” database; in order to find relevant articles, we combined each of the following keywords: “Fabry Disease,” “Endothelium,” “Vascular,” “Enzyme Replacement Therapy,” “Genetic Therapy,” “Molecular Chaperones.”

## 2. Pathophysiology

**Figure 1:**
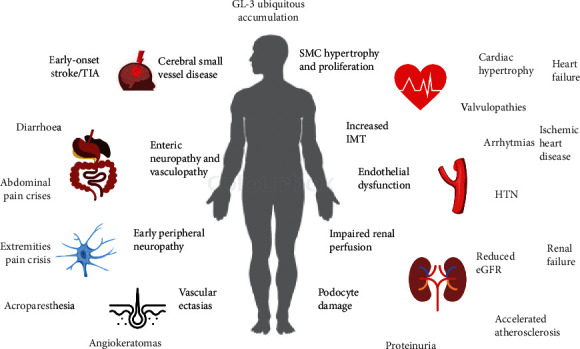
Pathophysiology and clinical correlates of Anderson–Fabry disease. The ubiquitous accumulation of GL-3 is central to disease onset and progression.

Accumulation of GL-3 begins in the fetal period [9, 10], but patients are asymptomatic during the first years of life. Initial symptoms depend on tissues and organs affected by the lysosomal accumulation of GL-3, indicating that the primary pathological processes take place inside the cells where substrate accumulation occurs. This phenomenon is thought to lead to structural damage and abnormal cell function (e.g., limited contractility of muscle cells, altered expression of surface molecules, or abnormal release of cellular products). In turn, it could trigger secondary pathological processes with demonstrated potential of systemic impact, including inflammation [11, 12], ischemia, hypertrophy, and fibrosis [13, 14]. Both primary and secondary pathological processes can progressively induce a damage in an organ system and contribute to multisystemic failure [13, 14] and frailty [15, 16]. The pathophysiology of Anderson–Fabry disease involves the ubiquitous accumulation of GL-3 in several cell types [9, 17]. The disease progression involves, over time, different organ systems. Late complications and failure may occur in the kidney, heart, or cerebrovascular system. Central and peripheral nervous system is usually involved first. This leads to several initial symptoms such as hypohidrosis, acroparesthesias, and episodic pain crises [9, 17] (Figure 1).

Ischemia plays a key role in shaping the disease phenotype, where small vessels in the cerebrovascular system, heart, kidney, peripheral nervous system, and skin can all be affected, reflecting the systemic vasculopathy that is typical of the disease [9, 17]. However, cerebrovascular complications (transient ischemic attacks and early-onset strokes) caused by cerebral vasculopathy are a major cause of morbidity and early mortality in patients with Anderson–Fabry disease, independent of gender [11, 18]. Other cardiovascular manifestations of the disease also include hypertension, left-ventricular hypertrophy, valvulopathies, and cardiac conduction disorders. Renal and heart failure, ischemic heart disease, and potentially fatal arrhythmias represent additional, life-threatening complications of the disease [5]. Several mechanisms are thought to contribute to ischemic tissue damage. Occlusion and luminal obstruction due to the accumulation of GL-3 in vascular endothelial cells, perturbation of the balance between vasodilators and vasoconstrictors, and thromboembolic complications could all play a role [13, 19].

Early peripheral neuropathy reflects the functional deterioration of neuronal cells in the peripheral autonomous and somatosensory nervous system, due to GL-3 deposits in the vasa vasorum of small myelinated and non-myelinated fibers [15, 20]. Deposits in the dorsal root ganglia cause abnormalities in the threshold of pain perception [17, 21]. Hypohidrosis occurs as a sign of selective damage to peripheral nerves [10, 18], although it has been attributed to lipid deposits in the small vessels surrounding the sweat glands as an alternative hypothesis [12, 19].

Early gastrointestinal manifestations are thought to be due to the accumulation of GL-3 in the vascular endothelium of the mesenteric blood vessels, in non-myelinated neurons, in perineural cells, and in autonomic ganglia of the gastrointestinal tract [14, 20].

Vascular cutaneous lesions (angiokeratomas) are caused by weakening of the capillary wall after the accumulation of GL-3 and the development of vascular ectasias in the dermis and epidermis [16, 21].

## 3. Anderson–Fabry Disease and Endothelial Dysfunction

**Figure 2:**
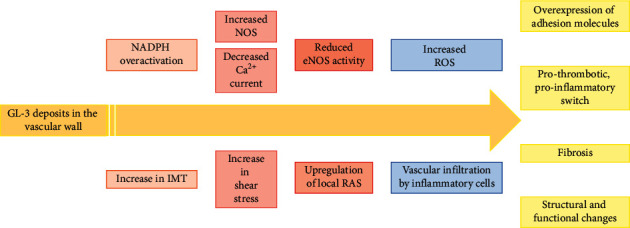
Possible mechanisms of endothelial dysfunction in Anderson–Fabry disease. IMT: intima-media thickness; NADPH: nicotinamide adenine dinucleotide phosphate; eNOS: endothelial nitric oxide synthase; RAS: renin-angiotensin system; RNS: reactive nitrogen species; ROS: reactive oxygen species.

Endothelial dysfunction in Anderson–Fabry disease has been described in several studies, both in terms of altered flow mediated dilation (FMD) and of serum biomarkers of dysfunctional endothelium [22]. Possible mechanisms behind this observation include accumulation of GL-3 in the endothelium; proliferation of smooth muscle cells; increase in the intima-media thickness (IMT); hyperexpression of endothelial activation markers; a phenotypic switch towards a prothrombotic phenotype; and decreased bioavailability of nitric oxide (NO) [23] (Figure 2). Specifically, GL-3 deposits in the vascular wall are thought to promote the proliferation of smooth muscle cells, causing remodelling of the arterial wall and the narrowing of the arterial lumen. This effect has also been described as a consequence of lyso-GL-3 accumulation [24]. The resulting increase in the shear stress might be responsible for a downstream cascade of molecular events, including the upregulation of local renin-angiotensin system that, in turn, induces a pro-thrombotic, pro-inflammatory status and impairs endothelial release of NO [25]. Another possibility is that the substrate accumulation itself is able to induce an overactivation of nicotinamide adenine dinucleotide phosphate (NADPH) and the uncoupling of endothelial NO synthase (eNOS), with lower NO bioavailability, increased formation of reactive oxygen species (ROS), and overexpression of cell adhesion molecules (CAMs) (i.e., intercellular adhesion molecule 1, ICAM-1; vascular cellular adhesion molecule 1, VCAM-1; and E-selectin) [26–28]. All these events might mediate the onset of vascular complications in Anderson–Fabry patients. This is in agreement with consistent evidence showing that the increase in ROS production plays a crucial role in the development of atherosclerosis and cardiovascular disorders. At the cellular level, in fact, ROS are able to induce irreversible damage to deoxyribonucleic acid (DNA), lipids, and proteins [29, 30], leading to low-density lipoprotein (LDL) oxidation, overexpression of adhesion molecules, and cellular dysfunction [31, 32].

ROS-induced transcription of CAMs, which is mediated by nuclear factor K-b (NF-Kb) and other transcription factors, also contributes to the Anderson–Fabry vasculopathy [33]. In fact, E-selectin, ICAM-1, and VCAM-1 induce the rolling and adhesion of leukocytes at the endothelial level, initiating the arterial wall infiltration and damage [28]. Notably, this effect is reversible after the administration of *α*-Gal A [28].

An additional mechanism of vascular dysfunction in the disease consists in GL-3-mediated internalization of calcium-activated potassium channels (KCa3.1), with consequent reduction in calcium currents and intracellular calcium levels and downregulation of eNOS [34]. Specifically, reduced KCa3.1 expression in the plasma membrane is secondary to clathrin-dependent lysosomal degradation of the channel, induced by the substrate accumulation [34] (Figure 2).

It has also been suggested that the accumulation of GL-3 alone is sufficient to dysregulate eNOS activity, with consequent decreased synthesis of NO and abnormal production of reactive nitrogen species, namely, 3-nitrotyrosine (3NT) [35] (Figure 2). Increased levels of 3NT were not only found in mice but also in biobanked plasma samples from patients with classical Anderson–Fabry disease, suggesting its potential use as a biomarker for vascular impairment in the disease [35]. According to preliminary evidence in humans, other molecules with the potential of serum biomarkers of the disease include matrix metalloproteinase 9 (MMP-9), angiostatin, symmetric dimethylarginine (SDMA), and the L-homoarginine (hArg)/SDMA ratio [36]. In particular, SDMA was found to be associated with diagnosed cardiomyopathy, indexed left-ventricular mass, and high sensitive troponin T in these patients [36]. MMP-9 and angiostatin levels were also elevated in patients compared to controls [36], potentially reflecting increased extracellular matrix turnover, although they might also mirror the reduced NO bioavailability typical of the disease [37]. Increased SDMA levels in association with reduced hArg/SDMA ratio among patients could be the expression of endothelial dysfunction and increased oxidative stress [38, 39]. Impairment in alternative, non-NO endothelium-dependent vasodilatory pathways has also been described in small human studies on normotensive and normocholesterolemic patients with Anderson–Fabry disease [40]. Specifically, compared with controls, patients showed increased vasodilation following acetylcholine infusion even after administration of the eNOS inhibitor NG-monomethyl-L-arginine (L-NMMA) [40]. The evidence of less vasoconstriction in patients compared with controls following L-NMMA infusion also suggests the dominance of alternative, non-NO pathways in patients with Fabry, possibly due to eNOS downregulation [40].

The accumulation of GL-3 in the lysosomes of vascular cells, and in particular in myocardiocytes in the cardiac phenotype, causes, in addition to the mechanisms described, alterations in energy metabolism, mitochondrial dysfunction, and activation of inflammatory molecules leading to autoimmune myocarditis [41–43].

In relation to the involvement of the mitochondrial dysfunction in the onset of oxidative stress, mutations in mitochondrial DNA (mtDNA) have been associated with a decline in energy production and an increased propensity for a number of pathological conditions. Specific mtDNA haplogroups may therefore lead to different mitochondrial and mtDNA vulnerability to oxidative stress, which might also be differently relevant in different tissues, thus modulating the phenotype and the natural course of the disease [44].

These pathological changes, associated with GL-3 related damage to the sympathetic nervous system, cause the onset of cardiac conduction disorders, left ventricular hypertrophy, and heart failure even with preserved ejection fraction [45, 46].

## 4. Enzyme Replacement Therapy and Emerging Treatment Options

**Figure 3:**
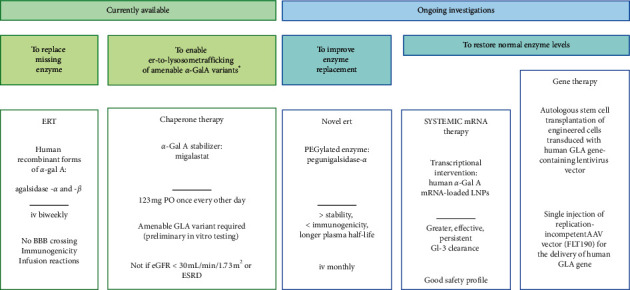
Enzyme replacement therapy and emerging alternatives. ^*∗*^abnormally folded, unstable *α*-Gal A protein with preserved enzymatic activity; iv: intravenous; ER: endoplasmic reticulum; eGFR: estimated glomerular filtration rate; ESRD: end-stage renal disease; BBB: blood-brain barrier; AAV: adeno-associated viral vector; LNPs: lipid nanoparticles.

The natural history of Anderson–Fabry disease was significantly improved by the introduction, in 2001, of the first specific treatment for the disease, i.e., ERT, based on the administration of human recombinant forms of *α*-Gal A [41, 42, 47, 48] (Figure 3).

In a healthy cell, the newly produced enzyme undergoes sequential post-translational modifications in the endoplasmic reticulum and Golgi apparatus; among the various modifications, the bond with mannose or mannose-6-phosphate (M6P) residues occurs in particular. The excreted enzymes can bind to a neighbor's M6P receptor cell with lysosomal disease (LD). The LD cell therefore internalizes the entire complex by endocytosis of the M6P receptor. The resulting endosomes containing the enzyme fuse with the lysosomes, providing the functioning enzyme in the LD cell and correcting the storage defect. This phenomenon is called cross correction and constitutes the point on which the lysosomal ERT is based [49]

There are currently two available ERT options: agalsidase-*α* (0.2 mg/kg/14 days) and agalsidase-*β* (1 mg/kg/14 days), which are administered intravenously [41, 42, 47, 48].

According to systematic reviews and meta-analyses, ERT stabilizes and may slow disease progression, especially when started at an early age, with evidence of a dose effect and benefits on major outcomes, such as cerebrovascular, cardiac, and renal complications [42–44, 48–50]. Specifically, a recent systematic literature review on 166 publications including 36 clinical trials examined the efficacy of ERT in adult men [42, 48]. In parallel to a drastic reduction in plasma, urine, and tissue concentrations of GL-3, ERT was found to determine a variety of clinical effects: from slowing the decline in estimated glomerular filtration rate (eGFR), to reducing or stabilizing cardiac remodelling and ameliorating neurological and gastrointestinal symptoms [42, 48]. The lack of clinical improvements, but also of further deterioration, during ERT was regarded as a marker of clinical benefit in an otherwise progressive, debilitating disease associated with a risk of premature mortality [42, 45, 46, 48, 51, 52].

An updated Cochrane review of 9 randomized controlled trials (RCTs) of agalsidase-*α* or -*β* compared to other or no interventions, for a total of 351 participants, showed significant improvement with ERT in terms of microvascular endothelial deposits of GL-3 and pain‐related quality of life, as well as positive effects on cardiac morphology and renal function, with a good safety profile and tolerability [43, 49]. However, no specific information was provided in the included trials on correlations of GL-3 with clinical events or survival, but these data would be better provided by patient registries, since long-term, large studies are required for this purpose [43, 49].

Further, a meta-analysis of 7 cohort studies and 2 RCTs involving 7513 participants (mean age 40.9 years, 51.9% men, about 20% on ERT, mean follow-up 4.1 years) and examining the benefit of ERT for stroke prevention showed lower stroke recurrence ratio in the ERT treatment group (8.2% versus 16%, *p*=0.03) [44, 50].

Overall, current evidence indicates that the most evident clinical results are obtained in subjects who started ERT at an earlier age [47–50, 53–56]. This was particularly true for renal events, where prevention of renal failure was only obtained if ERT was started before the development of glomerulosclerosis and proteinuria [49, 51, 55, 57]; otherwise, eGFR improvement was much more modest [48, 54]. For what concerns cardiac complications, subjects who began taking ERT before the development of cardiac fibrosis, i.e., substantially before the age of 30, were more likely to achieve a significant reduction in left ventricular mass; this effect was much more modest when ERT was started over 50 years of age [34, 52, 58]. Two recent publications have demonstrated a significant reduction in clinical events in patients who started ERT before age 40 compared to older ones [53, 54, 59, 60].

Despite the absence of head-to-head comparative studies between agalsidase-*α* and agalsidase-*β*, many studies show that ERT could improve outcomes in a dose-dependent manner. In studies where both recombinant enzymes were included (agalsidase-*α* 0.2 mg/kg/14 days and agalsidase-*β* 1 mg/kg/14 days), a greater reduction in plasma and urinary concentrations of GL-3 was found after treatment with agalsidase-*β* [55, 56, 61, 62]. These benefits were also obtained by increasing the usual dosage of agalsidase-*α*, with improvement in renal function and reduction of proteinuria [57, 58, 63, 64]. On the contrary, a reduction in the agalsidase-*β* dosage was associated with an increase in plasma, urinary, and renal values of GL-3, as well as with worsening of neurological and gastrointestinal symptoms [59–61, 65–67]; these negative effects were also found after switching to agalsidase-*α* [62, 68]. A large multicentre retrospective cohort study, in 2018, showed that agalsidase-*β* administration causes a greater biochemical response and a better reduction in left ventricular mass compared to agalsidase-*α* [69].

Recently, newer therapeutic options have risen to offer an alternative to ERT, overcome some ERT-related inconveniences, such as response variability, immunogenicity, infusion reactions, inability of blood-brain barrier crossing, and reproducibility of proper glycosylation patterns [63, 70], or attempt to intervene at the genetic or the transcriptional level to restore normal enzyme levels (Figure 3). They include oral chaperone therapy with migalastat [64, 65, 71, 72]; a novel PEGylated ERT, pegunigalsidase-*α* [66, 73, 74]; lucerastat, an inhibitor of the glucosylceramide synthetase (GCS) [75]; gene therapy using viral vectors [67–71, 74, 76–80]; and systemic messenger-RNA (mRNA) therapy (MRT), based on the delivery of biosynthetic mRNA transcripts as the source for therapeutic protein [63, 70]. Migalastat is an oral *α*-Gal A stabilizer representing an alternative to intravenous ERT, which facilitates normal lysosomal trafficking in the presence of susceptible mutated enzyme forms [64, 71]. Pegunigalsidase-*α*, a chemically modified *α*‐Gal A enzyme incorporating polyethylene glycol (PEG) moieties administered as an infusion directly into the bloodstream, is characterized by greater stability, reduced immunogenicity, and longer plasma half-life compared to traditional ERT, thus allowing monthly administration [66, 73]. Lucerastat is shown to effectively inhibit GCS and to markedly reduce the GL-3 substrate of the defective *α*-Gal A enzyme in Fabry subjects on ERT, independent of their mutation or phenotype [75]. Currently investigated gene therapies include the autologous stem cell transplantation of engineered cells (CD34+ cells transduced with the human GLA gene-containing lentivirus vector) and the delivery of a replacement copy of the missing gene by means of an adeno-associated viral vector (FLT190) [70, 72, 78, 81]. The monthly delivery of in vitro-synthesized human *α*-Gal A mRNA using a lipid nanoparticles- (LNP-) based formulation has been tested in a mouse model of the disease (GLAtm1kul) and in wild-type non-human primates, showing multiorgan biodistribution and deposition of the enzyme, effective and persistent (6 weeks) Gl-3 clearance that was superior to that obtained with ERT, and a good safety profile [63, 70, 73, 82]. If confirmed in human studies, this approach would represent another appealing opportunity to interfere with the natural history of the disease.

While migalastat has been recently approved for the treatment of Anderson–Fabry disease in the presence of specific mutations in the GLA gene and eGFR ≥30 mL/min/1.73 m^2^ [74, 83], safety and efficacy of the other therapeutic strategies are currently being investigated in phase 1 and 2 trials [66, 70, 71, 75].

Therapeutic recommendations emphasize that it is advisable to begin medical treatment of Fabry disease possibly before the onset of irreversible organ damage [84]; in particular, the treatment with ERT allows reducing the accumulation of GL3 and the initiation of events related to the endothelial dysfunction if started before the onset of tissue fibrosis and organ failure [85].

## 5. Conclusions

Anderson–Fabry disease is a rare, highly debilitating, inherited systemic disease where partial or total *α*-Gal A deficiency results in progressive multisystemic failure. Endothelial dysfunction represents one of the mechanisms behind the development of end-stage, irreversible complications of the disease, but the underlying mechanisms have only partially been elucidated. Unlike the past, promising therapies are now available or under investigation to delay the onset of organ damage and the related burden of morbidity and mortality. Early initiation of specific treatment offers unprecedented benefits in terms of protection against disease progression and related complications, but long-term, large, prospective studies are needed to define the relative impact on disease prognosis.

## Data Availability

No data were used to support this study.
